# Current practice and attitudes of stroke physicians towards rhythm-control therapy for stroke prevention: results of an international survey

**DOI:** 10.1186/s42466-023-00255-7

**Published:** 2023-07-06

**Authors:** Märit Jensen, Rustam Al-Shahi Salman, G. Andre Ng, H. Bart van der Worp, Peter Loh, Bruce C. V. Campbell, Jonathan M. Kalman, Michael D. Hill, Luciano A. Sposato, Jason G. Andrade, Andreas Metzner, Paulus Kirchhof, Götz Thomalla

**Affiliations:** 1 Department of Neurology, University Medical Center Hamburg-Eppendorf, Martinistraße 52, 20246 Hamburg, Germany; 2 German Centre for Cardiovascular Research (DZHK e.V.), Partner Site Hamburg/Kiel/Lübeck, Hamburg, Germany; 3 Centre for Clinical Brain Sciences, University of Edinburgh, Edinburgh, UK; 4 Department of Cardiovascular Sciences, University of Leicester, National Institute for Health Research Leicester Biomedical Research Centre, Glenfield Hospital, Leicester, UK; 5 Department of Neurology and Neurosurgery, Brain Center, University Medical Center Utrecht, Utrecht, The Netherlands; 6 Department of Cardiology, University Medical Center, Utrecht Heidelberglaan 100, Utrecht, The Netherlands; 7 Department of Medicine and Neurology, Melbourne Brain Centre at the Royal Melbourne Hospital, University of Melbourne, Parkville, VIC Australia; 8 The Florey Institute of Neuroscience and Mental Health, University of Melbourne, Parkville, VIC Australia; 9 Department of Cardiology, The Royal Melbourne Hospital, Melbourne, Australia; 10 Department of Clinical Neurosciences, Hotchkiss Brain Institute, University of Calgary, Calgary, AB Canada; 11 Department of Clinical Neurological Sciences, Western University, London, ON Canada; 12 Heart and Brain Laboratory, Western University, London, ON Canada; 13 University of British Columbia, Vancouver, BC Canada; 14 Montréal Heart Institute, Université de Montréal, Montréal, QC Canada; 15 Center for Cardiovascular Innovation, Vancouver, BC Canada; 16 Department of Cardiology, University Heart and Vascular Center Hamburg, University Medical Centre Hamburg-Eppendorf, 20246 Hamburg, Germany; 17 Institute of Cardiovascular Sciences, University of Birmingham, Birmingham, UK; 18 University of Melbourne, Melbourne, Australia

**Keywords:** Atrial fibrillation, Stroke, Rhythm-control therapy, Online survey

## Abstract

**Background:**

Patients with ischemic stroke and atrial fibrillation (AF) are at particularly high risk for recurrent stroke and cardiovascular events. Early rhythm control has been shown to be superior to usual care for the prevention of stroke and cardiovascular events for people with early AF. There are no data on the willingness to use rhythm control for patients with AF and acute ischemic stroke in clinical practice.

**Methods:**

An online survey was carried out among stroke physicians to assess current practice and attitudes toward rhythm control in patients with AF and acute ischemic stroke between December 22nd 2021 and March 24th 2022.

**Results:**

The survey was completed by 277 physicians including 237 from 15 known countries and 40 from unspecified countries. 79% (210/266) reported that they do not regularly apply treatment for rhythm control by ablation or antiarrhythmic drugs at all or only in small numbers (≤ 10%) of patients with AF and acute ischemic stroke. In those patients treated with rhythm-control therapy, antiarrhythmic drugs were used by the majority of respondents (89%), while only a minority reported using AF ablation (11%). 88% of respondents (221/250) stated that they would be willing to randomize patients with AF after acute ischemic stroke to either early rhythm control or usual care in a clinical trial.

**Conclusion:**

Despite its potential benefit, few patients with AF and acute ischemic stroke appear to be treated with rhythm control, which may result from uncertainty regarding potential complications of antiarrhythmic therapy in patients with acute stroke. Together with recent data on the effectiveness of early rhythm control in patients with a history of stroke, these results call for a randomized clinical trial to assess the efficacy of early rhythm control in patients with acute ischemic stroke and AF.

**Supplementary Information:**

The online version contains supplementary material available at 10.1186/s42466-023-00255-7.

## Background

Atrial fibrillation (AF), together with atherosclerotic disease, is one of the most common causes of ischemic stroke. There are more than 7.6 million incident cases of acute ischemic stroke worldwide per year, and about one in four of these patients have co-morbid AF [7]. Thus, there are more than 1.9 million new stroke patients with AF each year worldwide. Stroke patients with AF are at a high risk of developing recurrent stroke and other cardiovascular complications, such as myocardial infarction, heart failure, or cardiac death [2]. The randomized Early Treatment of Atrial Fibrillation for Stroke Prevention Trial (EAST-AFNET 4) demonstrated the benefit of early rhythm-control therapy in reducing cardiovascular events in patients with early AF [4]. This study initiated a paradigm shift in cardiology, with early rhythm control now being regularly applied for patients with newly diagnosed AF [1, 9]. The EAST-AFNET 4 study, however, did not include patients with acute ischemic stroke, a group of patients who are at especially high risk of recurrent stroke. While there is no doubt about the benefit of oral anticoagulation in stroke patients with AF, it is uncertain whether treatment that aims at restoring and maintaining sinus rhythm, i.e., rhythm control, is effective in preventing recurrent strokes and cardiovascular complications in these patients. Currently, international and national guidelines do not recommend rhythm-control therapy for acute stroke patients. There is uncertainty about the potential harms of antiarrhythmic therapy in patients with acute ischemic stroke, so treating physicians are more likely to refrain from antiarrhythmic treatment in these patients. In addition, cardiologists are reluctant to perform AF ablation early after stroke because patients with acute stroke are considered vulnerable to complications.

To evaluate current clinical practice and attitude towards rhythm control in patients with acute ischemic stroke and AF, we conducted an international survey among stroke physicians.

## Methods

A survey questionnaire was created using Survey Monkey (http://www.surveymonkey.com). The survey comprised 13 questions (Additional file 1): five to define characteristics of respondents; six to establish their current practice regarding rhythm control in patients with AF and acute ischemic stroke; and two to assess participants’ attitude towards a randomized clinical trial on rhythm control in these patients.

The online survey was opened 22nd December 2021 and the link to the survey was distributed via the following networks: the German Stroke Trials Network (GSTN), the National Institutes of Health Stroke Trials Network (NIH StrokeNet), the British and Irish Association of Stroke Physicians (BIASP), the Health Research Board Stroke Clinical Trials Network, Ireland (HRB–SCTNI), the Canadian Stroke Network, the Belgian Network for Clinical Stroke Trials (BeNET), stroke networks in the Netherlands and Spain, and via the newsletter of the Early versus Late initiation of direct oral Anticoagulants in post-ischaemic stroke patients with atrial fibrillatioN (ELAN) trial. Network members were encouraged to further disseminate the survey to stroke physicians in their hospitals or regional networks. The survey was closed on 24th March 2022.

The results of the completed online survey were downloaded, and descriptive statistics were produced for each question.

## Results

### Characteristics of respondents

**Fig. 1:**
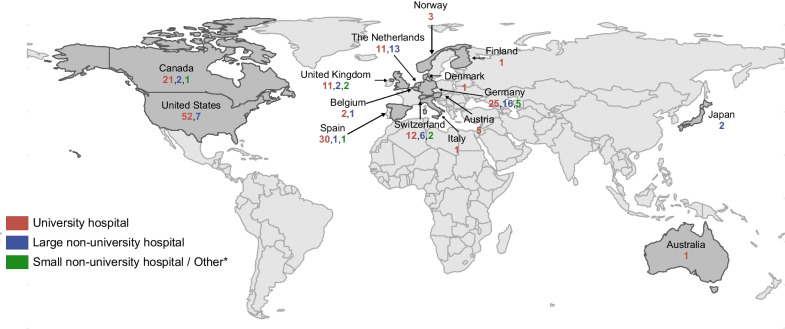
World map representing the distribution of the 277 respondents across 15 countries which are further grouped into physicians working in university hospitals, large non-university hospitals and small non-university hospitals. *Not working in a hospital (e.g., practice). The country of 40 participants is unspecified

A total of 277 clinicians completed the survey, of whom 254 (92%) were neurologists, 5 (2%) were cardiologists, and 18 (7%) were other physicians involved in stroke care (Table 1). The majority of responders (161; 58%) had more than ten years of experience in their speciality and were working in university hospitals (207; 75%). 237 responders came from 15 specified countries around the world, and 40 from unspecified countries (Fig. 1). United States of America (59; 21%), Germany (46; 17%) and Spain (32; 12%) were the three countries with most respondents to the survey.

**Table 1:** Characteristics of participants

Characteristics of participants	Total participants (n = 277)
Speciality	
Neurology/Stroke physicians	254 (92%)
Cardiology	5 (2%)
Other involved in treatment of stroke patients	18 (7%)
Level of experience	
Junior (< = 5 years)	53 (19%)
Senior (6–10 years)	63 (23%)
Expert (> 10 years)	161 (58%)
Type of hospital	
University hospital	207 (75%)
Large non-university hospital/tertiary hospital	59 (21%)
Small non-university hospital	10 (4%)
Other (e.g., practice)	1 (< 1%)
Specialized stroke unit available	259/267 (97%)
Consulting cardiologist available	267/268 (> 99%)

### Organisation of acute stroke care within participants’ institutions

**Fig. 2:**
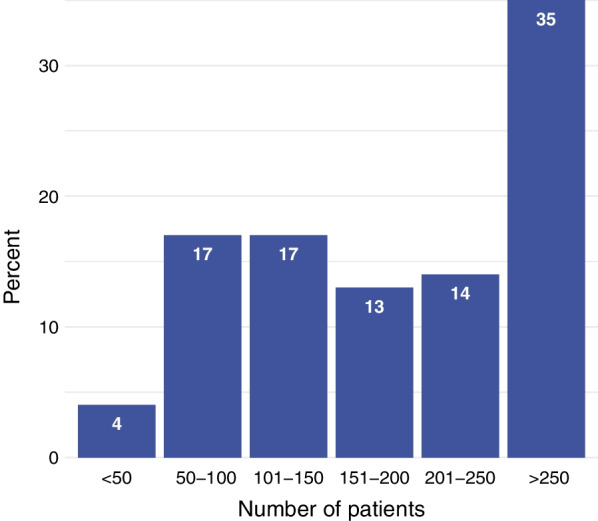
Estimated number of patients with acute ischemic stroke and atrial fibrillation treated per year. The question was answered by 268 participants

Virtually all participants (259/267; 97%) answered that their hospital had a specialized stroke unit. All but one respondent reported that a consultant cardiologist was available. Most responders (212/268; 79%) stated that they treated more than 100 patients with acute ischemic stroke and AF per year, and more than one third treating even over 250 of these patients per year (95/268; 35%) (Fig. 2).

### Current use of rhythm-control therapy among physicians

**Fig. 3:**
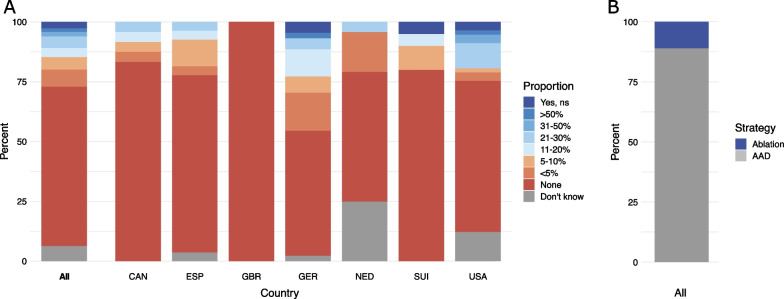
**A** Estimated proportion of patients with acute stroke and atrial fibrillation receiving treatment for rhythm control within 4 weeks of stroke onset. **B** Primary treatment strategy for rhythm control. The underlying questions were answered by 266 participants. Countries with < 10 responses (Australia, Austria, Finland, Denmark, Italy, Belgium, Japan, Norway) and unspecified countries are not shown individually. ns, not specified; AAD, antiarrhythmic drugs

Two thirds of participants (177/266, 67%) do not regularly treat patients with acute ischemic stroke and AF using rhythm-control therapy by ablation or antiarrhythmic drugs (Fig. 3A). Another 12% (33/266) use rhythm control only in less than 5% or in 5 to 10% of patients. There were considerable differences between respondents from different countries. Physicians from the United Kingdom reported that no acute stroke patients received therapy for rhythm control, while in Germany 18% of physicians reported using rhythm-control therapy in more than 10% of patients. Among the patients treated with rhythm control, antiarrhythmic drugs were used by the majority of respondents (89%), in contrast to only 11% reporting the use of AF ablation (Fig. 3B). The response pattern varied by country, ranging from 15% AF ablation in the United States and 16% in Switzerland to only 5% AF ablation in Spain (Additional file 1: Table S1). Among 254 stroke physicians, only 5% stated that EAST-AFNET 4 results had changed their clinical practice.

### Attitude towards randomization into a clinical trial of early rhythm control after acute stroke

221 of 250 participants (88%) answered that they would be willing to randomize patients with acute ischemic stroke and AF into a clinical trial comparing early rhythm-control therapy (by either ablation or antiarrhythmic drugs) to usual care. The majority (202/219; 92%) stated that antiarrhythmic drugs would be the first strategy to perform early rhythm control in their institution.

## Discussion

Treatment for rhythm control is reported to be used only rarely in patients with AF and acute ischemic stroke. Stroke physicians appear to be largely unaware of the findings from the EAST-AFNET 4 trial or do not relate these results to their clinical practice of treating patients with acute ischemic stroke and AF. The survey also illustrates the need for a controlled trial of early rhythm-control therapy in patients with AF and an acute stroke.

EAST-AFNET 4 demonstrated that a strategy of systematic early rhythm control using antiarrhythmic drugs or ablation reduces the incidence of stroke and cardiovascular mortality in patients with newly diagnosed AF (< 12 month before randomization) compared to usual care [4]. This has initiated a paradigm shift in clinical preference towards rhythm control rather than rate control as the preferred strategy for patients with newly diagnosed AF. In a subgroup analysis of the EAST-AFNET 4 trial of patients with a history of stroke, the treatment effect of early rhythm control in patients with a prior stroke was consistent with the findings of the primary analysis [3]. There were no safety concerns regarding the use of early rhythm control in patients with a history of stroke. The treatment effect appeared stronger than in patients without a history of stroke suggesting that patients with acute ischemic stroke and AF may benefit particularly from early rhythm control. Observational data from a large Korean database comprising over 50,000 patients lend further support to the benefit of rhythm control in patients with AF and a history of stroke. In patients with a prior stroke, rhythm-control therapy started within one year after a diagnosis of AF was associated with a lower risk of recurrent stroke than usual care [5].

These findings are promising because patients with AF and a history of stroke represent a clinically vulnerable population, and any intervention that reduces the burden of adverse cardiovascular outcomes in this group could have a substantial benefit. They also align with a separate subanalysis of the EAST-AFNET 4 trial identifying a stronger treatment effect in patients with AF and a high comorbidity burden (CHA2DS2-VASc ≥ 4) [8]. Patients with a recent acute ischemic stroke constitute a population at even higher risk, as the risk of recurrent ischemic stroke as well as of other adverse cardiovascular events is highest within the first few weeks after stroke [6]. However, concerns regarding potential side effects or complications may limit the use of rhythm control, which may be one of the reasons for the low rates of use among stroke physicians suggested by our survey. This appears to apply especially to AF ablation, as only around 10% of patients would be treated with ablation as first-line therapy according to the responses in our survey. Notably, there were differences between countries, with the United States and Switzerland indicating a higher propensity to use ablation for patients with acute stroke and AF as compared to other countries.

Overall, our results suggest that data from a randomized trial are needed to resolve the uncertainty about potential harms of antiarrhythmic treatment in the early phase of stroke.

The vast majority of stroke physicians participating in our survey were willing to randomize stroke patients with AF patients in a clinical trial of early rhythm control. This may reflect the perception that the evidence for early rhythm control in AF is sufficient to suggest a potential benefit, but not sufficient to change clinical practice for acute ischemic stroke patients.

There are limitations to our survey. We do not know the response rate, given that the survey was sent to national and international stroke networks and further disseminated by the recipients. Furthermore, over half of the respondents came from four countries only, and results may not be applicable to international practice or representative of stroke physicians in other parts of the world. We also did not ask about the reasons why physicians refrain from using rhythm-control therapy in acute stroke patients, so we can only speculate about this. Finally, we cannot exclude a response bias which might favour stroke physicians interested in this treatment option in participating in this questionnaire so that the results might overestimate awareness and a positive attitude to rhythm-control therapy for AF. Thus, the knowledge and acceptance of rhythm-control therapy for patients with stroke in clinical practice may be even lower than reported. Due to the study design, we cannot rule out the possibility that more than one participant from each centre contributed data to the questionnaire.

## Conclusion

In summary, our study suggests reluctance to use rhythm control in the management of patients with acute stroke and AF, despite evidence of its effectiveness in reducing cardiovascular events in patients with AF and cardiovascular risk factors. This reluctance likely results from uncertainty about possible complications of antiarrhythmic treatment in patients with acute stroke. Stroke physicians are receptive to the idea of randomizing such patients in a clinical trial of early rhythm-control therapy, the Early treatment of Atrial fibrillation for Stroke prevention Trial in acute Stroke (EAST-STROKE) (NCT05293080).

## Supplementary Information

**Additioanl file 1**. Supplementary material.

## Data Availability

The datasets used and/or analysed during the current study are available from the corresponding author on reasonable request.

## Abbreviations

AF: Atrial fibrillation
BeNET: Belgian Network for Clinical Stroke Trials
BIASP: British and Irish Association of Stroke Physicians
EAST-AFNET 4: Early Treatment of Atrial Fibrillation for Stroke Prevention Trial
EAST-STROKE: Early treatment of Atrial fibrillation for Stroke prevention Trial in acute Stroke
ELAN: Early versus Late initiation of direct oral Anticoagulants in post-ischaemic stroke patients with atrial fibrillation
GSTN: German Stroke Trials Network
HRB–SCTNI: Health Research Board Stroke Clinical Trials Network, Ireland
NIH StrokeNet: National Institutes of Health Stroke Trials Network
