# Supplementary material for: Current practice and attitudes of stroke physicians towards rhythm-control therapy for stroke prevention: results of an international survey
Source: Neurol Res Pract. 2023 Jul 6;5:29. doi: 10.1186/s42466-023-00255-7 (PMC10324107; doi:10.1186/s42466-023-00255-7)
Supplement: Supplementary file 1 — Additioanl file 1. Supplementary material. [file 42466_2023_255_MOESM1_ESM.pdf]

Supplementary Material

Current practice and attitudes of stroke physicians towards rhythm-control therapy for stroke prevention: Results of an international survey

Märit Jensen[1,2*], Rustam Al-Shahi Salman[3], G Andre Ng[4], H Bart van der Worp[5], Peter Loh[6], Bruce Campbell[7,8], Jon M Kalman[9], Michael D Hill[10], Luciano A. Sposato[11,12], Jason G Andrade[13], Andreas Metzner[2,14], Paulus Kirchhof[2,14,15], Götz Thomalla[1]

[1] Department of Neurology, University Medical Center Hamburg-Eppendorf, Hamburg, 20246, Germany

[2] German Centre for Cardiovascular Research (DZHK e.V.), partner site Hamburg/Kiel/Lübeck, Germany

[3] Centre for Clinical Brain Sciences, University of Edinburgh, Edinburgh, UK

[4] Department of Cardiovascular Sciences, University of Leicester, National Institute for Health Research Leicester Biomedical Research Centre, Glenfield Hospital, Leicester

[5] Department of Neurology and Neurosurgery, Brain Center, University Medical Center Utrecht, the Netherlands

[6] Department of Cardiology, University Medical Center Utrecht Heidelberglaan 100, Utrecht, The Netherland

[7] Department of Medicine and Neurology, Melbourne Brain Centre at the Royal Melbourne Hospital, University of Melbourne, Parkville, Victoria, Australia

[8] The Florey Institute of Neuroscience and Mental Health, University of Melbourne, Parkville, Victoria, Australia

[9] Department of Cardiology, The Royal Melbourne Hospital, Melbourne, Australia

[10] Department of Clinical Neurosciences, Hotchkiss Brain Institute, University of Calgary, Calgary, Alberta, Canada

[11] Department of Clinical Neurological Sciences, Schulich School of Medicine and Dentistry Western University London, Ontario, Canada

[12] Heart & Brain Laboratory Western University London, Ontario, Canada

[13] University of British Columbia, Vancouver, British Columbia, Canada; Montréal Heart Institute, Université de Montréal, Montréal, Québec, Canada; Center for Cardiovascular Innovation, Vancouver, British Columbia, Canada

[14] University Heart and Vascular Center Hamburg, Department of Cardiology, University Medical Centre Hamburg-Eppendorf, Hamburg, 20246, Germany

[15] Institute of Cardiovascular Sciences, University of Birmingham, Birmingham, UK

UKE HAMBURG

Rhythm control in stroke patients with atrial fibrillation

Welcome to our survey

In preparation for a study on early rhythm control in patients with AF and acute ischemic stroke, we are attempting to assess the current clinical practices related to this through a short questionnaire.
Your participation in this survey is voluntary and anonymous. The data collected will be kept confidential and will only be used for research purposes. Your responses will help us gain insights into the current state of clinical practice in early rhythm control for patients with AF and acute ischemic stroke.
The survey will take approximately 5 minutes to complete. Your participation is highly appreciated, and we thank you in advance for your time and contribution to this study.
If you have any questions or concerns regarding the survey, please do not hesitate to contact us.

Contact details:
Dr. Märit Jensen
Department of Neurology
University Medical Center Hamburg-Eppendorf, Hamburg, Germany
m.jensen@uke.de

UKE HAMBURG

Rhythm control in stroke patients with atrial fibrillation

General information

1. Which country are you from?

2. Which specialty are you?

- Neurology/Stroke physician
- Cardiology
- Other

3. Which level of experience do you have in your specialty?

- Junior (<=5 years)
- Senior (5-10 years)
- Expert (>10 years)

4. Which type of hospital are you working in?

- University hospital
- Large non-university hospital/tertiary hospital
- Small non-university hospital
- Other/I am not working in any hospital

Rhythm control in stroke patients with atrial fibrillation

Acute stroke care at your hospital

5. Does your hospital have a specialized stroke unit?

- ◯ Yes
- ◯ No

6. How many patients with **acute ischemic stroke and atrial fibrillation** are treated at your hospital per year (estimated)?

- ◯ <50
- ◯ 50-100
- ◯ 101-150
- ◯ 151-200
- ◯ 201-250
- ◯ >250

7. Do you have a consulting cardiologist available at your hospital?

- ◯ Yes
- ◯ No

Rhythm control in stroke patients with atrial fibrillation

Considering your treatment of acute stroke patients with atrial fibrillation:

8. Do you regularly perform early rhythm control (i.e., specific treatment to restore sinus rhythm) by ablation or antiarrhythmic drugs (e.g., Amiodarone, Dronedarone, Flecainide) in these patients?

- ◯ Yes
- ◯ No
- ◯ I don't know

UKE HAMBURG

Rhythm control in stroke patients with atrial fibrillation

Considering all patients with acute ischemic stroke and atrial fibrillation:

9. Which proportion of these patients are regularly treated with early rhythm control (i.e., ablation or antiarrhythmic drugs like dronedarone) within the first 4 weeks of stroke onset at your hospital?

- ◯ <5%
- ◯ 5-10%
- ◯ 11-20%
- ◯ 21-30%
- ◯ 31-50%
- ◯ >50%

10. To be more specific: for which proportion of these patients (i.e. patients receiving early rhythm control) is ablation the primary strategy for early rhythm control (instead of antiarrhythmic drugs)?

0% ——— 100% [ ]

11. Have the results of EAST-AFNET4 changed your clinical practice?

- ◯ Yes
- ◯ No
- ◯ I don't know the study results

UKE HAMBURG

Rhythm control in stroke patients with atrial fibrillation

12. Would you be willing to randomize patients with acute ischemic stroke and atrial fibrillation to early rhythm control (by either ablation or antiarrhythmic drugs) vs. usual care in a clinical trial?

- ◯ Yes
- ◯ No

Rhythm control in stroke patients with atrial fibrillation

13. What would be your first strategy to perform early rhythm control in acute stroke patients in your hospital?

- ◯ Antiarrhythmic drugs
- ◯ Ablation

Supplementary Table S1: Estimated proportion of patients with acute stroke and atrial fibrillation receiving treatment for rhythm control with 4 weeks of stroke onset and primary treatment strategy for early rhythm control.

		**Rhythm control for patients with acute stroke and AF within 4 weeks after stroke (proportion)**									**Primary treatment strategy**	
**Country**	**N**	**Don't know**	**None**	**< 5%**	**5-10%**	**11-20%**	**21-30%**	**31-50%**	**> 50%**	**Yes, ns**	**AAD**	**Ablation**
All	266	6%	67%	7%	5%	4%	5%	2%	2%	3%	89%	11%
United States	57	12%	63%	4%	2%	0%	11%	4%	2%	4%	85%	15%
Germany	44	2%	52%	16%	7%	11%	5%	0%	2%	5%	92%	8%
Spain	27	4%	74%	4%	11%	4%	4%	0%	0%	0%	95%	5%
Canada	24	0%	83%	4%	4%	4%	4%	0%	0%	0%	92%	8%
The Netherlands	24	25%	54%	17%	0%	0%	4%	0%	0%	0%	94%	6%
Switzerland	20	0%	80%	0%	10%	5%	0%	0%	0%	5%	84%	16%
United Kingdom	14	0%	100%	0%	0%	0%	0%	0%	0%	0%	-	-

The underlying questions were answered by 266 participants. Countries with <10 responses (Australia, Austria, Finland, Denmark, Italy, Belgium, Japan, Norway) and unspecified countries are not shown individually. Abbreviations: ns, not specified; AAD, antiarrhythmic drugs.
